# Atg4b-Dependent Autophagic Flux Alleviates Huntington’s Disease Progression

**DOI:** 10.1371/journal.pone.0068357

**Published:** 2013-07-08

**Authors:** Catia C. Proenca, Natacha Stoehr, Mario Bernhard, Shanon Seger, Christel Genoud, Ana Roscic, Paolo Paganetti, Shanming Liu, Leon O. Murphy, Rainer Kuhn, Tewis Bouwmeester, Ivan Galimberti

**Affiliations:** 1 Developmental and Molecular Pathways, Novartis Institutes for Biomedical Research, Basel, Switzerland; 2 Novartis Pharma AG Basel, Switzerland; 3 Friedrich Miescher Institute Basel, Switzerland; National University of Singapore, Singapore

## Abstract

The accumulation of aggregated mutant huntingtin (mHtt) inclusion bodies is involved in Huntigton’s disease (HD) progression. Medium sized-spiny neurons (MSNs) in the corpus striatum are highly vulnerable to mHtt aggregate accumulation and degeneration, but the mechanisms and pathways involved remain elusive. Here we have developed a new model to study MSNs degeneration in the context of HD. We produced organotypic cortico-striatal slice cultures (CStS) from HD transgenic mice mimicking specific features of HD progression. We then show that induction of autophagy using catalytic inhibitors of mTOR prevents MSNs degeneration in HD CStS. Furthermore, disrupting autophagic flux by overexpressing Atg4b in neurons and slice cultures, accelerated mHtt aggregation and neuronal death, suggesting that Atg4b-dependent autophagic flux influences HD progression. Under these circumstances induction of autophagy using catalytic inhibitors of mTOR was inefficient and did not affect mHtt aggregate accumulation and toxicity, indicating that mTOR inhibition alleviates HD progression by inducing Atg4b-dependent autophagic flux. These results establish modulators of Atg4b-dependent autophagic flux as new potential targets in the treatment of HD.

## Introduction

Huntington’s disease (HD) is a fatal, autosomal dominant inherited neurodegenerative disease that classically occurs with a triad of movement disorder, cognitive decline and psychiatric symptoms. The clinical symptoms of HD are mainly due to massive death of medium sized-spiny neurons (MSNs) in the corpus striatum [1]–[3]. The hallmark of MSNs degeneration is the appearance of aggregated mutant huntingtin (mHtt) inclusion bodies, decline of dopaminergic signaling (e.g. loss of DARPP-32) and neuronal death [4], [5]. Selective degeneration of MSNs causes an imbalance in the cortico-striatal-thalamocortical circuit which is thought to be the cause of chorea and cognitive decline characteristic of HD [6]. Thus, prevention of MSNs degeneration is thought to be critical to alleviate the hyperkinetic and cognitive deteriorations observed in HD and modeling MSNs degeneration in a native disease-relevant circuit context (such as cortico-striatal slices) represents a unique opportunity to study disease relevant pathways. Indeed, increasing evidence has indicated that organotypic brain slices maintain features of neuronal circuits over weeks *in vitro* and exhibit synaptic and structural plasticities as *in vivo* [7]–[9].

Macroautophagy (hereafter referred to as autophagy) is an intracellular turnover pathway through which cytosolic components, such as damaged or pathological proteins and organelles, are engulfed by a double-membraned autophagosome vesicle, which fuses with the lysosome, leading to cargo degradation [10]–[12]. Lipidation of microtubule-associated protein light-chain 3 (LC3) is an indicator of the formation of autophagosome/autolysosomes and defines the turnover of autolysosomes or autophagic flux. Atg4b is a cysteine protease that together with Atg5/Atg7 mediates the lipidation of all LC3 isoforms, therefore representing a central node in the regulation of the autophagic flux. Although LC3 lipidation is not induced in alternative pathways of autophagy, it is considered essential for Atg5/Atg7-dependent conventional autophagy and overexpression of Atg4b blocks conventional autophagy by reducing lipidation of all LC3 isoforms [13]–[15].

The mechanistic target of rapamycin (mTOR) is known to negatively regulate autophagy [16]. Allosteric mTOR inhibitors (e.g. rapamycin and analogs) mediate clearance of mHtt fragments and protect against mHtt-induced toxicity in non-neuronal cells [17]–[20]. However, recent studies showed that RAD001, an allosteric mTOR inhibitor, was inefficient in preventing neurodegeneration in the R6/2 mouse model [21], [22]. Thus, it is still unclear whether inhibition of the mTOR pathway can prevent specifically MSNs degeneration by inducing neuronal autophagy. Interestingly, catalytic mTOR inhibitors were recently shown to be more potent at inducing autophagic flux and reducing mHtt accumulation in non-neuronal cells as compared to allosteric mTOR inhibitors, suggesting that ATP competitive mTOR inhibition may induce stronger neuronal autophagic flux [20], [23].

Here we evaluated whether Atg4b-dependent autophagic flux prevents HD-associated MSNs degeneration by targeting mHtt degradation. We established CStS from two HD mouse models (R6/2 and Hdh^(CAG)150^) to assess if autophagy induction ameliorates MSNs degeneration. We first showed that R6/2 and Hdh^(CAG)150^ CStS recapitulate early and late signs of HD-associated MSNs degeneration and represent an accessible system to evaluate selected paradigms *in vitro*. Treatment of R6/2 and Hdh^(CAG)150^ CStS with a catalytic mTOR inhibitor, AZD8055 yielded a reduction of accumulated mHtt and an amelioration of MSNs degeneration. On the other hand, overexpression of Atg4b increased mHtt accumulation in cortical neurons and under this condition AZD8055-treatment was inefficient, suggesting that AZD8055 reduces mHtt levels by inducing Atg4b-dependent autophagic flux. Taken together our results have highlighted the importance of developing new agents to target Atg4b-dependent autophagic flux that could be used for treatment of HD and other neuronal proteinopathies.

## Results

### MSNs Degeneration is Recapitulated in HD CStS

**Figure 1:**
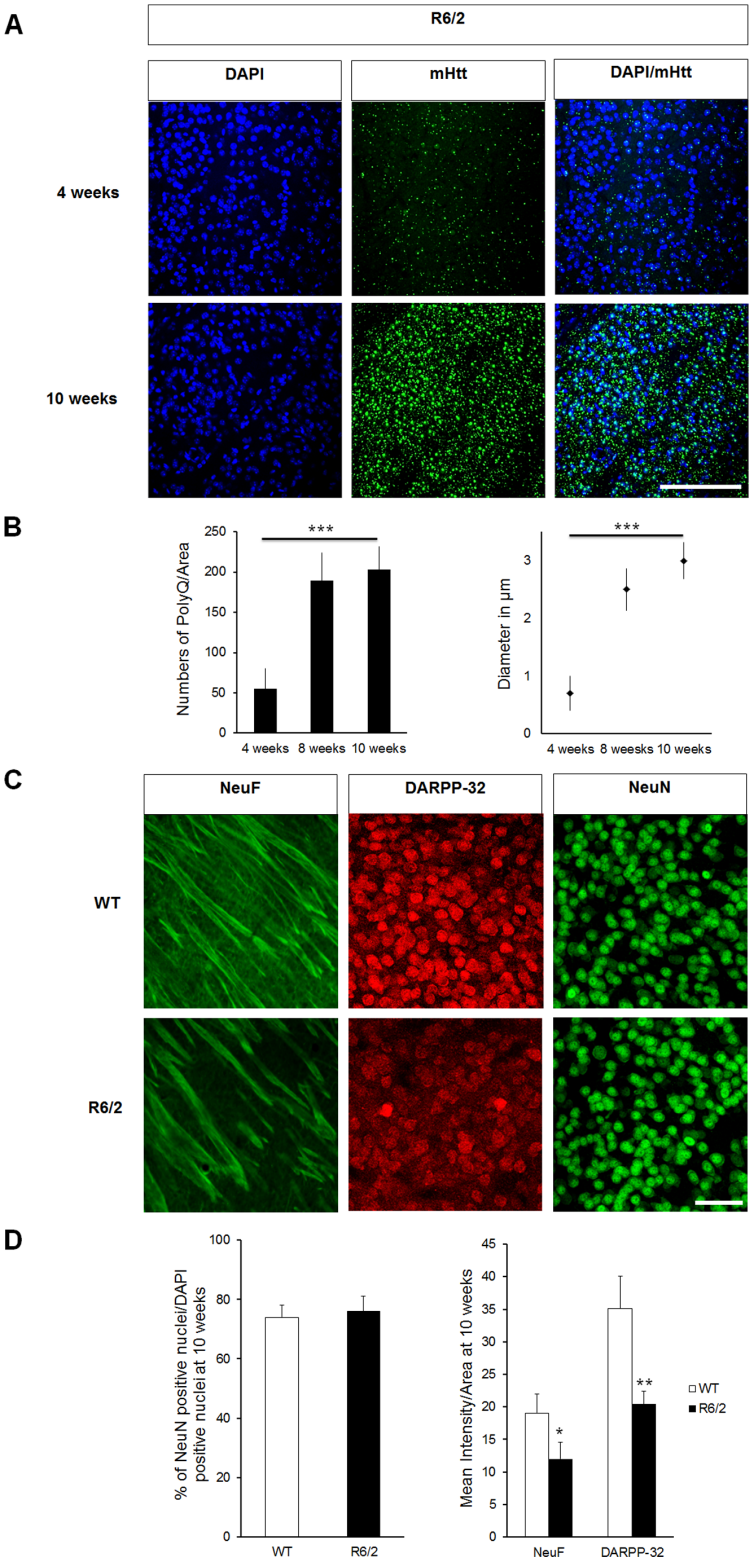
MSNs degeneration in R6/2 mice. A) Immunohistochemistry against DAPI (blue) and mHtt (green) of R6/2 brain sections shows progressive mHtt accumulation in striatum. B) Quantification of mHtt staining from A) showing mHtt aggregation in terms of density (left) and size (right). R6/2 mice develop progressive mHtt aggregation. C) Progressive striatal neurodegeneration in R6/2 mice. Brain sections from adult R6/2 mice and WT controls were stained for a striatal marker DARPP-32 (red), neurofilament (NeuF, green) and NeuN (green) at 10 weeks of age. By this time the R6/2 mice have developed mHtt accumulation and they have decreased DARPP-32 staining as well as NeuF, whereas the neuronal marker NeuN is unchanged. D) Left: the ratio of striatal NeuN and DAPI positive nuclei is unchanged between WT and R6/2 mice at 10 weeks of age, in line with the limited cell death characteristic of the R6/2 mouse model. Right: quantification of C) shows a highly significant decrease in both NeuF and DARPP-32 intensity. Three mice per condition; N = 5 images/each; median values ± SEM; Students t test:*p<0.05, **p<0.01 Bars and ***p<0.001: (A) 200 µm, (C) 50 µm.

MSNs degeneration is a central event in HD progression and is characterized by neuronal atrophy, decreased DARPP-32 and neurofilament (NeuF) proteins, as well as the appearance of mHtt accumulation [1]. To quantify HD-dependent MSNs degeneration, we used the R6/2 mouse model. These mice express human exon1 fragment of Htt with 148 to 153 CAG repeats and display an aggressive HD phenotype recapitulating disease progression in just a few weeks [24], [25]. We observed a progressive increase of the aggregate density and size in brain sections from R6/2 mice, with inclusions reaching a diameter of up to 3 µm at 10 weeks (Fig. 1A and 1B). We also detected a reduction of neuronal processes that paralleled a decrease of striatal DARPP-32 levels, suggesting that MSNs are in an advanced stage of degeneration by 10 weeks of age in R6/2 mice (−30% ±2.6 for NeuF and −40% ±2.1 for DARPP-32, Fig. 1C and 1D). No difference in the distribution and ratio of NeuN positive cells/DAPI positive cells were observed at 10 weeks of age (WT 74±4 and R6/2 76±5, Fig. 1C and 1D), confirming that the decrease of striatal DARPP-32 levels are not an unspecific consequence of cell death [26], [27].

**Figure 2:**
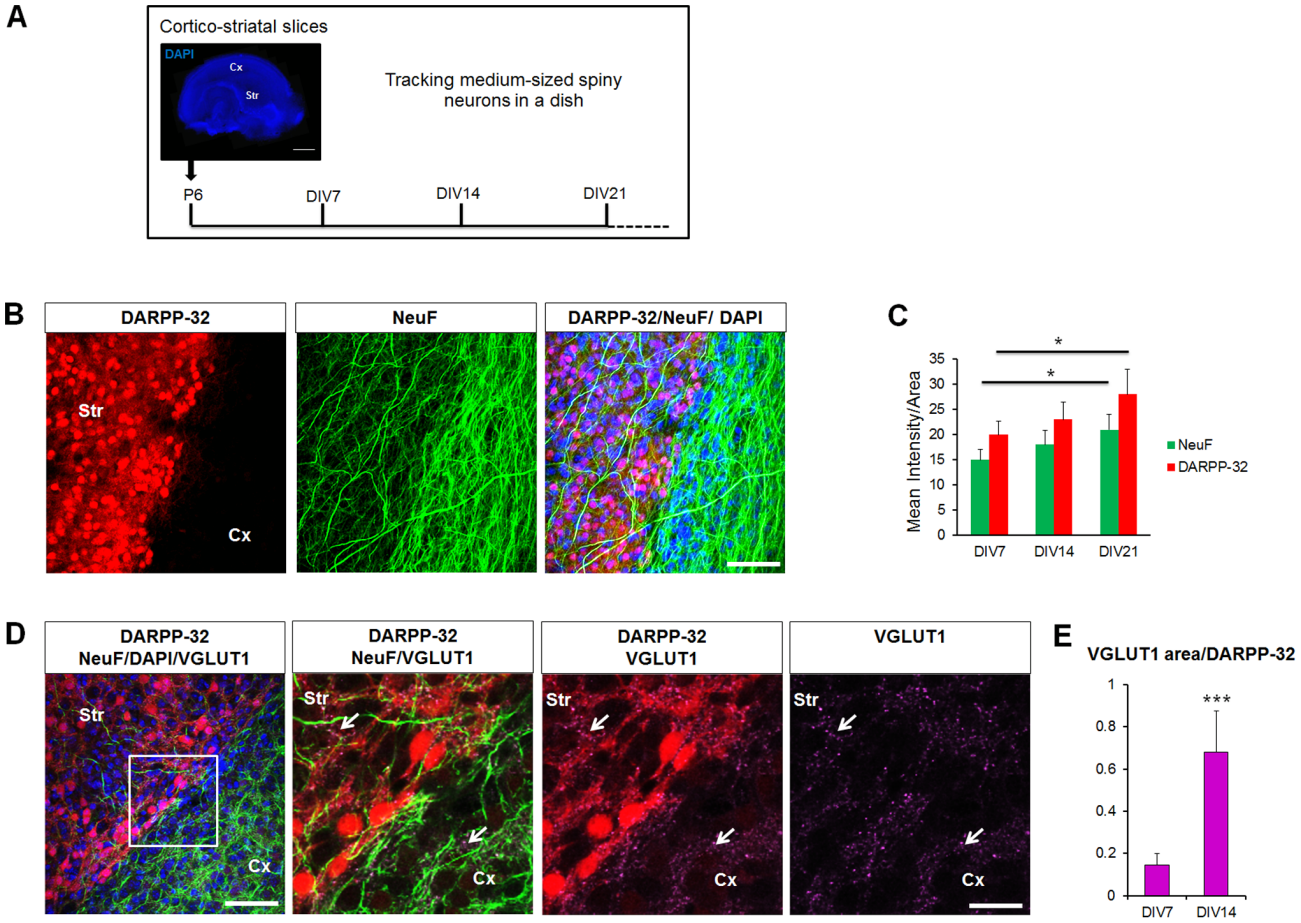
MSNs can be cultured for weeks in organotypic cortico-striatal slice cultures. A) Schematic of the preparation for the oganotypic cortico-striatal slice cultures used in this study. Cortico-striatal slices (CStS) were prepared at postnatal day 6 (P6) and maintained for several weeks *in vitro*. For time course analysis, CStS were typically collected at DIV7, DIV14 and DIV21. B) Analysis of cortico-striatal slices prepared from P6 WT mice and maintained in culture for 4 weeks (DIV28). Immunohistochemistry with neuronal markers DARPP-32 (red) and NeuF (green) show strong stainning in the striatum and cortex, respectively. C) Quantification of DARPP-32 and NeuF intensity in WT CStS. Note, a progressive increase over time *in vitro*. D) Left: representative single confocal plane for the immunohistochemistry of CStS with neuronal markers DARPP-32 (red), NeuF (green), VGLUT1 (magenta) and DAPI (blue) at DIV7. Right: zoom in highlighting VGLUT1 positive glutamate vesicles (arrows) within the cortex and striatum. E) Quantitative analysis of VGLUT1 area normalized to DARPP-32 at DIV7 and DIV14. Note how VGLUT1 significantly increases with development from DIV7 to DIV14. Str = Striatum; Cx = Cortex; N = 10 images from 5 independent slices; median values ± SEM; Students t test:*p<0.05 and ***p<0.001 Bars: (A) 1 mm, (B, D left) 200 µm, (D right) 40 µm.

**Figure 3:**
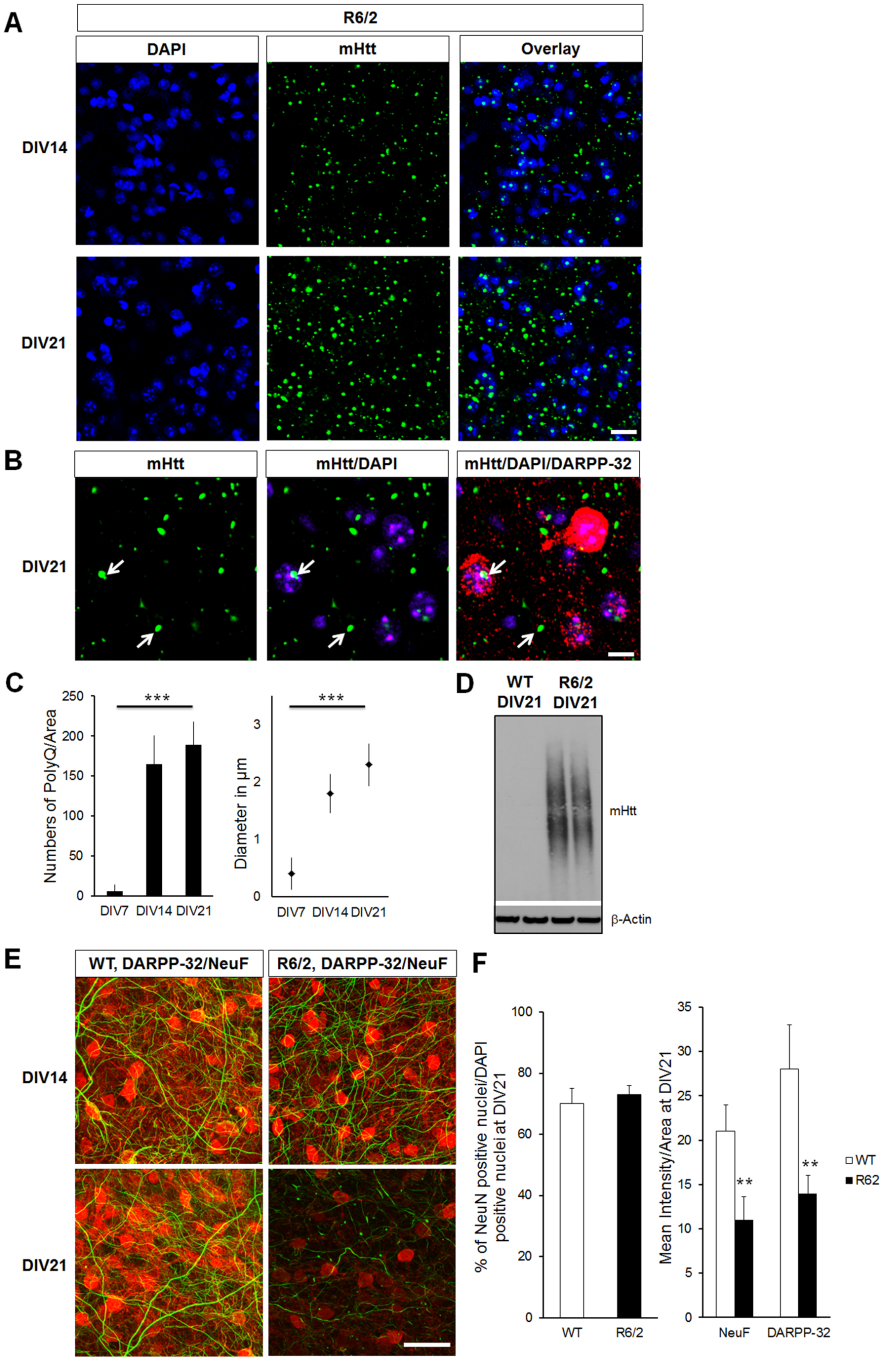
CStS recapitulate MSNs degeneration observed in R6/2 mouse model. A) Single confocal planes for the immunohistochemistry of CStS derived from R6/2 show progressive mHtt accumulation in the striatum at DIV14 and DIV21. B) Higher magnification of MSNs in R6/2 slices visualized with DAPI (blue), DARPP-32 (red) and mHtt (green) at DIV21. Note the presence of cytosolic and nuclear mHtt accumulation (arrows). C) Quantification of mHtt density (left) and size (right) throughout development. Aggregates are primarily increasing in size after DIV14 and density stabilizes. D) Biochemical detection of mHtt accumulation using AGERA at DIV21. 10 µg from the total lysate of WT and R6/2 slices were loaded and selective signals were detected in R6/2 slices; each lane represents an aliquot of a distinct slice. N = 5 independent slices. E) Selective degeneration of MSNs in R6/2 slices visualized by immunohistochemistry with the striatal marker DARPP-32 (red) and cortical NeuF (green). Neurodegeneration is observed as a decrease in marker intensity. F) Left: quantification for the ratio of striatal NeuN and DAPI positive nuclei in WT and R6/2 slices at DIV21. Right: quantification of DARPP-32 and NeuF intensity per area in WT and R6/2 slices at DIV21. N = 10 images from 5 independent slices; median values ± SEM; Students t test: **p<0.01 and ***p<0.001 Bars: (A) 30 µm, (B) 10 µm, (D) 25 µm.

A major challenge in HD is to understand why given the ubiquitous expression of mHtt throughout the body, is there a selective degeneration of cortical and striatal neurons. Several studies have focused on studying defects caused by overexpressed mHtt in non-neuronal cells and it had added value to our understanding of cell toxicity [11]. However, models where we can directly address these mechanisms in the cells selectively affected in HD are still lacking. To overcome this challenge, we have established a novel organotypic cortico-striatal slice culture (CStS) model in which neuronal viability and function can easily be addressed (Fig. 2A). This approach allows a longitudinal analysis of defined neuronal circuits and maintains for weeks *in vitro,* organotypic features of distinct brain regions such as the ones affected in HD: cortex and striatum (Fig. 2B) [7]–[9], [28]. We found that DARPP-32 and NeuF levels in striatum of WT slices progressively increase with time from DIV7 to DIV21, paralleling the neuronal maturation observed *in vivo* (Fig. 2C). Moreover, VGLUT1 positive vesicles were found in striatum and increased over time *in vitro*, indicating that cortical glutamatergic afferents not only innervate the striatum but develop over time in our slice preparation (Fig. 2D and Fig. 2E). We then analyzed if CStS from R6/2 mice would develop neurodegeneration *in vitro*, similar to the one observed *in vivo*. Strikingly, we observed that the striatum of CStS from R6/2 developed accumulation of mHtt throughout DIV 7–21, with cytosolic and nuclear inclusions measuring up to 2 µm in diameter at DIV21 and present in both DARPP-32 positive MSNs and DARPP-32 negative cells, in line with the characteristic distribution of mHtt inclusions in the R6/2 mouse model that have been shown to be in both MSNs and interneurons (Fig. 3A, 3B and 3C) [29]. Accumulation of mHtt was also detected by Agarose gel electrophoresis for resolving aggregates (AGERA, Fig. 3D). We then analyzed the degeneration of MSNs in R6/2 slices, and found a significant decrease of DARPP-32 and NeuF in R6/2 slices as compared to WT, (−40% ±3.2 and −43% ±2.4, respectively; Fig. 3E and 3F). Similarly to Figure 1D, no difference in the ratio of NeuN positive cells/DAPI positive cells were observed at DIV21 (WT 70±5 and R6/2 73±3, Fig. 3F). Our results demonstrate that the R6/2 CStS recapitulate mHtt-associated MSNs degeneration in few weeks *in vitro* and thus can be used to study manipulations to disease onset and progression.

**Figure 4:**
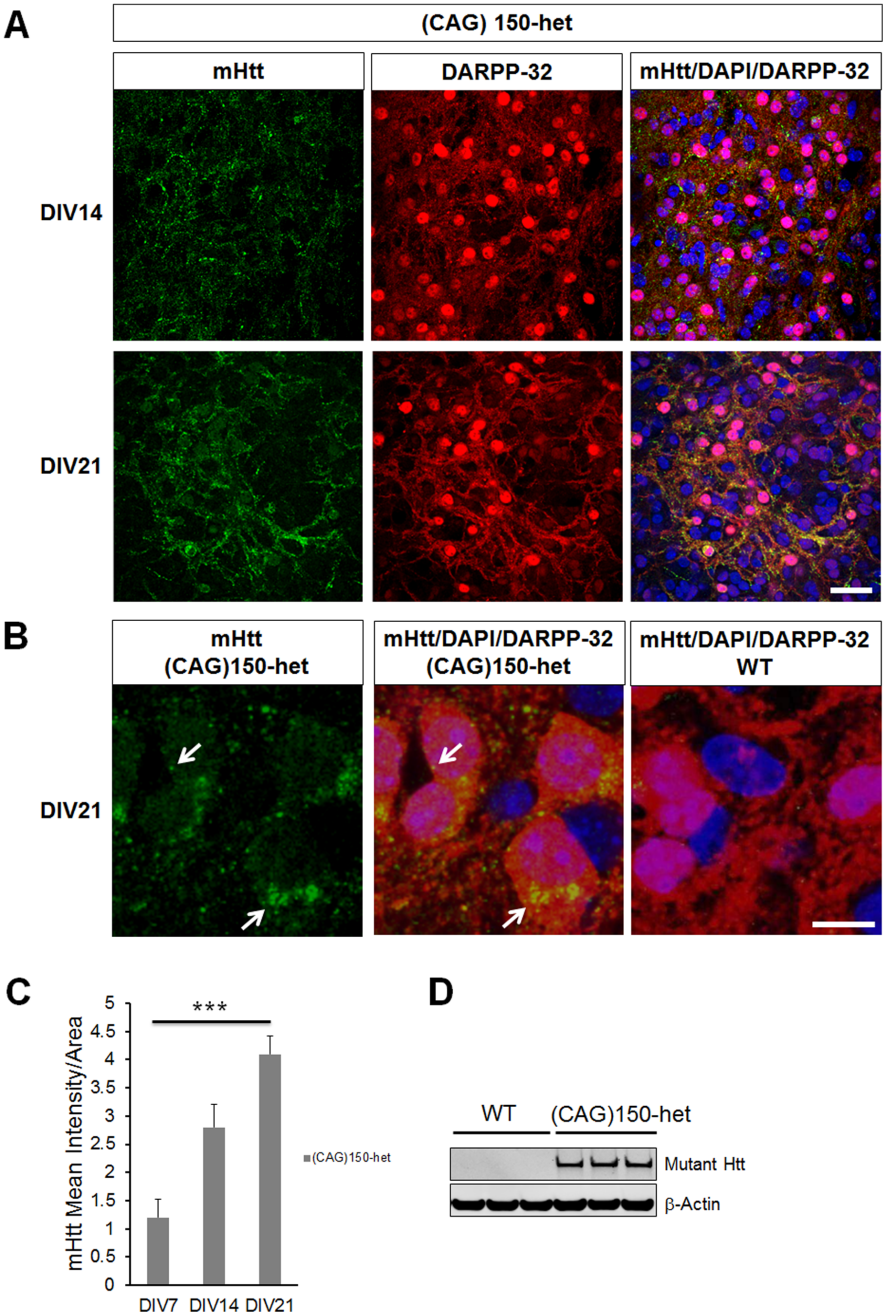
CStS recapitulate MSNs degeneration observed in (CAG)150-het mouse model. A) Single confocal planes for the immunohistochemistry of CStS derived from (CAG)150-het mice show progressive mHtt accumulation in the striatum at DIV14 and DIV21. B) Higher magnification of MSNs in (CAG)150-het slices show selective mHtt accumulation at DIV21. Note the extranuclear mHtt accumulation and few small nuclear inclusions (arrows). C) Time course quantification of mHtt intensity per area shows progressive accumulation in (CAG)150-het slices. D) Western blot showing mHtt is selective to (CAG)150-het slices. Biochemical detection of soluble mHtt in (CAG)150-het slices; each lane represents an aliquot of 10 µg from total lysates of distinct slices. N = 10 images from 5 independent slices; median values ± SEM; ***p<0.001 Bars: (A) 30 µm, (B) 10 µm.

To strengthen our findings in slices we analyzed a second HD mouse model that expresses endogenous full-length mutant Huntigntin with a CAG expansion in the murine Htt gene, the Hdh^(CAG)150^ mouse model. In contrast to R6/2, these mice represent a model comparative to the human condition because of the slow accumulation of toxic nuclear mHtt inclusions. In fact, nuclear mHtt inclusions start to appear at approximately 7 months of age and loss of DARPP-32 levels start to appear only at 25 months [25]. Interestingly, we found a progressive increase in mHtt accumulation in MSNs of CStS from Hdh^(CAG)150^ heterozygous (HET) (Fig. 4A, Fig. 4C). This accumulation appeared to be primarily extranuclear with few small nuclear inclusions at DIV21 (Fig. 4B). Moreover, soluble full-length mHtt was selectively detected in HET Hdh^(CAG)150^ CStS using a specific antibody against the longer polyQ epitope (Fig. 4D). Given the slow progression of MSNs degeneration in this model, DARPP-32 and NeuF levels were not significantly different between WT and HET Hdh^(CAG)150^ CStS (data not shown), indicating that HET Hdh^(CAG)150^ CStS exhibit early signs of the ongoing MSNs degeneration and can be used to assess mechanisms that can prevent the appearance of nuclear mHtt inclusions. Taken together these results demonstrate that HET *Hdh* ^(CAG)150^ mutant and R6/2 CStS recapitulate HD-dependent MSNs degeneration *in vitro* and can be used as an unique model to study and manipulate disease progression at different points in the neuronal population selectively affected in HD.

### mTOR Inhibition Stimulates Autophagic Flux in Neurons

**Figure 5:**
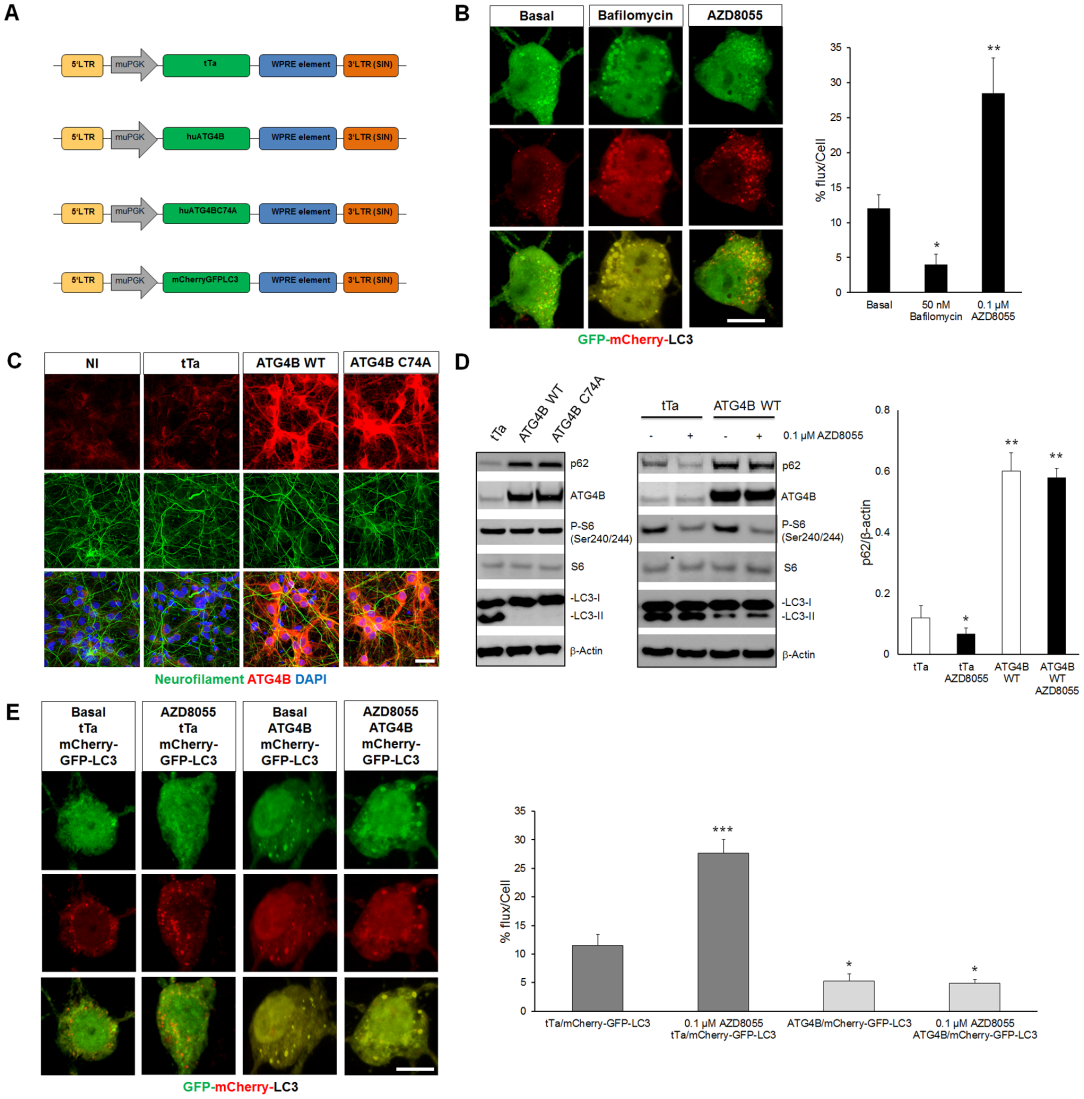
AZD8055, an mTOR inhibitor stimulates neuronal autophagy by inducing Atg4b-dependent autophagy flux in cultured neurons. A) Schematic representation of the lentiviral backbones used to measure neuronal autophagy flux. tTa was used as a control, mCherryGFPLC3 to measure autophagy flux by monitoring the different fluorescent signal and Atg4b WT or mutant (C74A) were used to block autophagy. B) Left: representative images for the detection of neuronal autophagy flux using mCherry-GFP-LC3 in cultured cortical neurons. Bafilomycin was used as negative control as it blocks acidification of autophagosome, blocking autophagy flux. Right, quantification of mCherry-GFP-LC3 shows how AZD8055 significantly increases autophagy flux (see materials and methods for the quantification of the autophagy flux). C) Representative images of cultured cortical neurons non-infected (NI) or transduced with tTa, Atg4b WT or Atg4b C74A. Note the similar Neurofilament (green) distribution in the different conditions, indicating no toxic impact for the Atg4b WT and Atg4b C74A overexpression (red). D) Left: biochemical analysis of LC3 autophagy markers for the conditions in (C). Both Atg4b WT and Atg4b C74A affected LC3 lipidation (LC3-II form) and induced p62 accumulation. Right: biochemical analysis of the effective mTOR inhibition and p62 degradation upon AZD8055 treatment. Note how the AZD8055-dependent p62 degradation is abolished once Atg4b WT is overexpressed. Far right: quantification of p62 normalized to β-actin shows a significant increase of p62 level upon Atg4b overexpression, as compared with cells transduced with tTa. AZD8055 treatment led to a significant increase in autophagy flux observed by a decrease in p62 level. However it was inefficient in the presence of Atg4b overexpression. E) Left: Representative images of cultured cortical neurons transduced with tTa/mCherry-GFP-LC3 or Atg4b/mCherry-GFP-LC3 with and without AZD8055 treatment. Right: quantitative analysis for neuronal LC3 autophagy. AZD8055 significantly increased LC3 autophagy flux in tTa/mCherry-GFP-LC3 neurons and did not have an effect in Atg4b/mCherry-GFP-LC3 neurons. N = 50 images from 3 independent preparations; median values ± SEM; Student’s t test: *p<0.05, **p<0.01 and ***p<0.001 Bars: (B and E) 10 µm, (C) 30 µm.

Blockade of the mTOR pathway stimulates autophagy and it has been shown to reduce accumulation of mHtt in non-neuronal cells [16], [20], [30]. However, if similar mechanisms are implicated in neurons is still unclear. To address this question, we made use of the recently developed imaging-based assay that takes advantage of the different sensitivities that GFP and mCherry have to pH. In this assay, cells are transduced with a tandem fluorescent tagged mCherry-GFP-LC3 construct. GFP reporters lose their fluorescence upon reaching the acidic environment of the autolysosome, whereas mCherry is relatively stable. Thus, an increase in red signal is a direct consequence of increased autophagosome-lysome fusion, giving a good indication of autophagic flux [31]. To test if the mTOR pathway can modulate autophagy specifically in neurons, we transduced cultured cortical neurons with the mCherry-GFP-LC3 construct and treated them with a catalytic inhibitor of the mTOR pathway (AZD8055). Bafilomycin, an H^+^-ATPase inhibitor that prevents acidification of autolysosome was used as a negative control as it blocks autophagosome/lysosome fusion reducing autophagic flux [23], [32], [33]. We found that neuronal autophagic flux was 12% ±4 at basal condition and after bafilomycin treatment it was 3 fold lower (4% ±1.5) with clustering of autophagosomes as previously described in non-neuronal-cells [23], [31]. Remarkably, we observed that AZD8055 increased autophagy flux by 2.7 fold (28.5% ±4.8) indicating that catalytic mTOR inhibition is a potent inducer of neuronal autophagic flux (Fig. 5B).

To dissect the downstream autophagy pathway targeted by AZD8055, we affected LC3 lipidation by overexpressing Atg4b WT or a protease activity-deficient mutant, Atg4b C74A. Both have been shown to prevent ATG7 from binding to LC3, abolishing LC3 conjugation to *phosphatidylethanolamine (*PE) as well as to deconjugate LC3 from PE, leading to autophagic structures which are defective in the final closing step and thus blocking autophagy [14], [15]. Indeed, we found that overexpression of either Atg4b WT or C74A led to p62 accumulation (a long-lived protein known to be degraded by conventional autophagic flux), a sharp decline of LC3-II and defective autophagic structures without affecting the neuronal organization visualized with the neurofilament distribution (Fig. 5C, 5D and 5E). Moreover, AZD8055 reduced p62 protein levels in tTa control neurons and was inefficient in preventing the p62 accumulation in Atg4b neurons (tTa 0.12±0.04, tTa AZD8055 0.067±0.02, Atg4b 0.6±0.06, Atg4b AZD8055 0.58±0.03), demonstrating that AZD8055 leads to p62 degradation in an Atg4b-dependent manner (Fig. 5D). In the same line, when we measured autophagic flux induced by AZD8055 in the presence of Atg4b overexpression, we also observed that affecting LC3 lipidation prevented AZD8055-mediated effects quantified in Figure 5B (Fig. 5E), thus suggesting that Atg4b acts downstream of mTOR and it is critical to modulate neuronal autophagic flux.

### Catalytic mTOR Inhibition Rescues MSNs Degeneration in HD Cortico-striatal Slice Cultures

**Figure 6:**
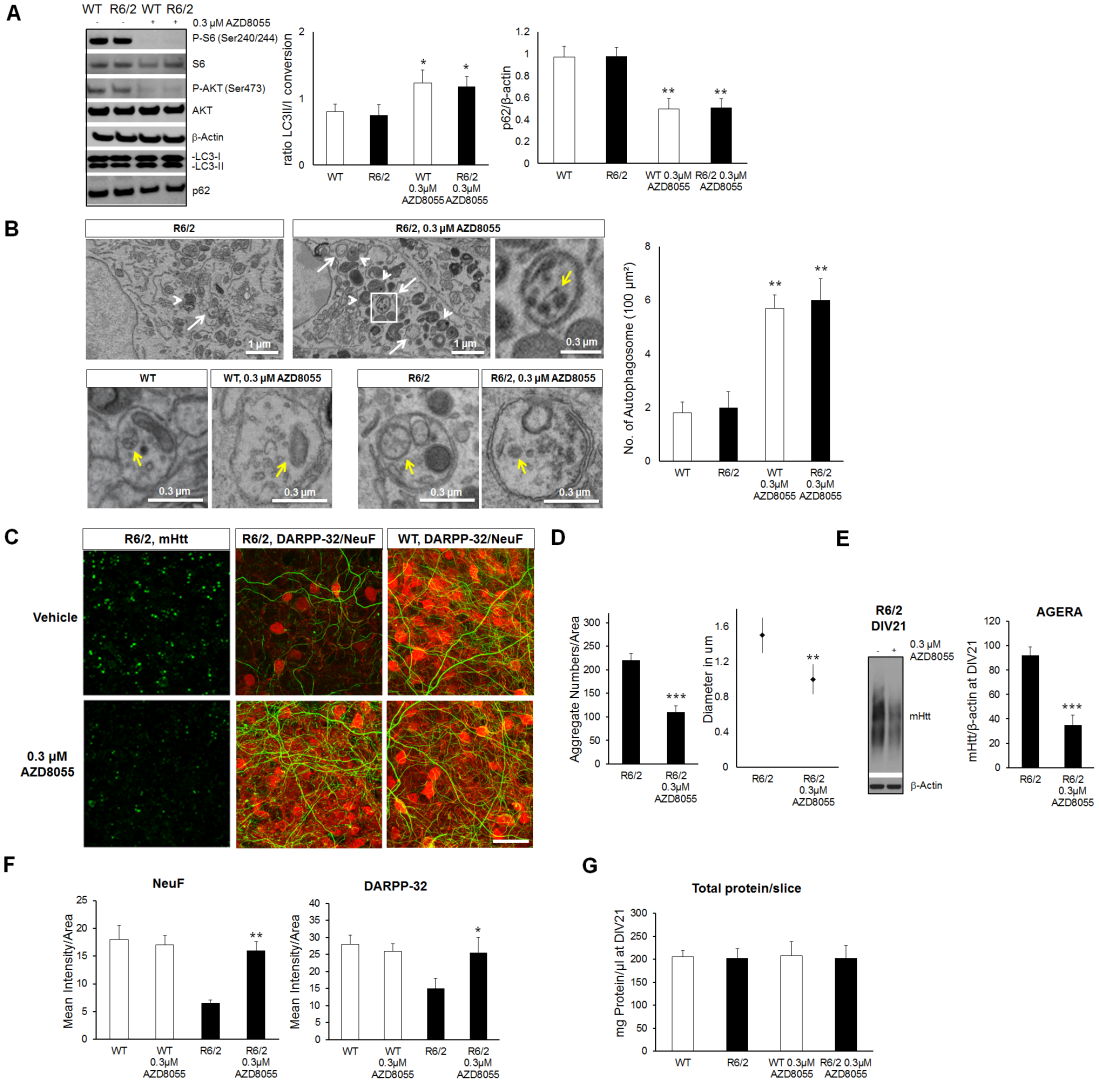
Activation of autophagy via mTOR inhibition rescues HD phenotype in CStS from R6/2 mice. A) Effective mTOR inhibition and autophagy induction in CStS upon AZD8055 treatment at DIV21. Left: biochemical detection of downstream signaling molecules in the mTOR pathway show how phospho-S6 (P-S6) and phospho-Akt (P-Akt) are efficiently blocked upon AZD8055 treatment. Biochemical detection of LC3 and p62 show how AZD8055 is inducing autophagy in brain slices by increasing the LC3 conversion and by decreasing p62 levels. Right: quantification of LC3II/LC3I conversion ratios and p62 levels in the AZD8055-treated and non-treated condition. Note how AZD8055 is efficiently inducing autophagy in both WT and R6/2 slices. B) Electron microscopy (EM) of representative WT and R6/2 brain slices treated with AZD8055 from DIV14 to DIV16. Up: autophagosomes (white arrows) and autolysosomes (white arrowheads) in AZD8055 R6/2 untreated and treated slices. Note the zoom of one autophagosome containing different structures (yellow arrow). Down: representative autophagosomes containing cytosolic contents (yellow arrows) in WT and R6/2 slices with and without AZD8055 treatment. Right: quantification of the number of autophagosome/100 µm^2^ indicates an increase upon AZD8055 treatment. C) Representative images of R6/2 and WT slices that were stained with mHtt (green), DARPP-32 (red) and NeuF (green). AZD8055 treatment led to obvious decrease in mHtt staining as well as amelioration in DARPP-32 distribution. D) Quantification of mHtt aggregation from C). AZD8055 treatment led to a reduction of striatal mHtt aggregate number as well as size. E) Quantification of mHtt levels in total lysates of DIV21 R6/2 slices treated with AZD8055 or with vehicle by AGERA. F) Quantification of C) shows that AZD8055 leads to a rescue of DARPP-32 and NeuF levels. G) Total protein levels were not changed by mTOR inhibition with AZD8055 in DIV21 slices. N = 10 images from 5 independent slices; median values ± SEM; Student’s t test: *p<0.05, **p<0.01 and ***p<0.001 Bar: (B) 1 µm and 0.3 µm, (C) 30 µm.

**Figure 7:**
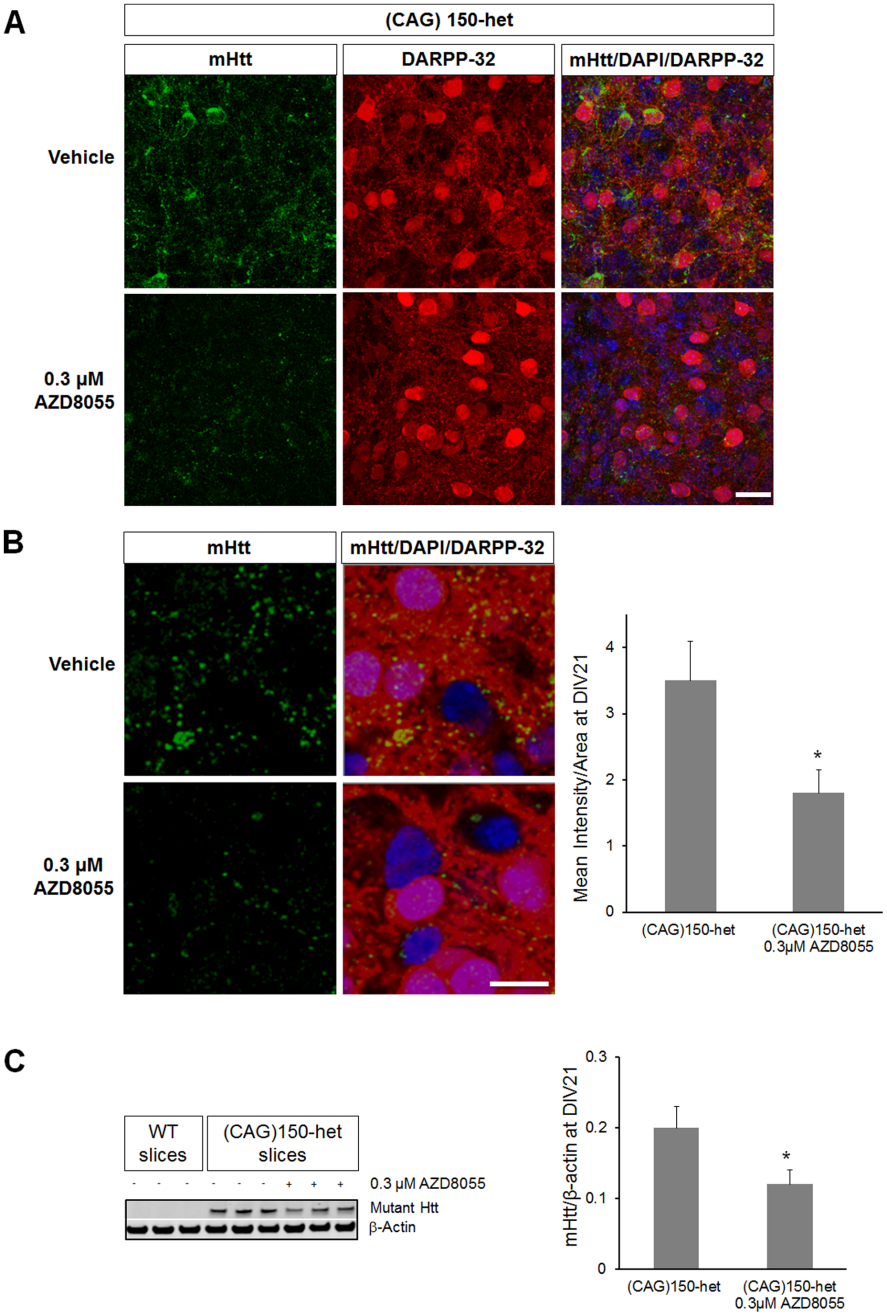
AZD8055 reduces mHtt levels in (CAG)150-het slices. A) Single confocal planes for the immunohistochemistry of (CAG)150-het slices against mHtt (green), DARPP-32 (red), DAPI (blue) and treated with AZD8055 or vehicle at DIV21. Note, how AZD8055 treatment leads to a reduction of striatal mHtt accumulation in (CAG)150-het slices. B) Left: higher magnification of MSNs in (CAG)150-het slices visualized with DAPI (blue), DARPP-32 (red) and mHtt (green) at DIV21. Right: quantification of mHtt mean intensity. AZD8055 leads to a significant reduction of mHtt intensity. C) Western blot analysis of (CAG)150-het slices treated with AZD8055 also show a significant reduction in mHtt levels which is quantified on the right. N = 10 images from 5 independent slices; median values ± SEM; Student’s t test: *p<0.05 Bars: (A) 20 µm, (B) 10 µm.

We then analyzed if catalytic inhibition of the mTOR pathway could be used to rescue MSNs degeneration. We started by analyzing the effects of AZD8055 in CStS slices by measuring phosphorylation of mTOR substrates. We tested different concentrations and time treatments and observed that a 7-day treatment with AZD8055 at 0.3 µM led to a strong reduction of P-S6 (Ser240/244) and P-AKT (Ser 473) at DIV21 (Fig. 6A left). We then measured LC3II/I ratio as an indicator of autophagy. LC3II/I ratio was similar in CStS from WT and R6/2 mice suggesting that basal autophagy is similar at this stage (Fig. 6A right). Treatment with AZD8055 significantly increased LC3II/I ratio as an indication of increased autophagy (from basal 0.82±0.17 to 1.22±0.15 Fig. 6A right). In addition, AZD8055 reduced p62 protein levels in both WT and R6/2 slices (WT 0.97±0.1, R6/2 0.98±0.08, WT AZD8055 0.5±0.09, R6/2 AZD8055 0.51±0.08, Fig. 6A right), indicating its efficacy in degrading long-lived proteins such as p62 in brain slices. We then assessed the increase of autophagosomes in brain slices upon AZD8055 treatment by electron microscopy (EM). We found that AZD8055 significantly increased the number of autophagosomes/100 µm^2^ in both WT and R6/2 slices (WT 1.8±0.4, R6/2 2±0.6, WT AZD8055 5.7±0.5, R6/2 AZD8055 6±0.8, Fig. 6B). Interestingly, autophagosomes were found to contain a number of cytosolic contents in both WT and R6/2 slices, suggesting no failures in cargo recognition in R6/2 slices (Fig. 6B). Subsequently we tested the ability of AZD8055 to rescue mHtt-dependent MSNs degeneration in R6/2 CStS. First, we measured mHtt aggregation in slices treated with AZD8055 or vehicle and found that compound treatment led to a significant reduction in density and size of mHtt aggregates in striatal cells, in addition to decreasing insoluble mHtt (about 50%; Fig. 6C, Fig. 6D and Fig. 6E). Moreover, AZD8055 also rescued the levels of DARPP-32 and NeuF in R6/2 CStS, to levels close to WT (Fig. 6C and Fig. 6F). Notably, the total protein concentration of single CStS at DIV21 was similar in treated and non-treated conditions (around 200 ng), indicating that AZD8055 did not lead to a decrease of global protein levels (Fig. 6G). We further characterized the efficiency of AZD8055 in preventing MSNs degeneration by treating CStS from the *Hdh* ^(CAG)150^ HET mouse model. Interestingly, we found that compound treatment decreased endogenous mHtt accumulation to half in striatal cells at DIV21 as measured by immunohistochemistry (*Hdh* ^(CAG)150^ 3.5±0.6, *Hdh* ^(CAG)150^ with 0.3 µM AZD8055 1.8±0.3, Fig. 7A, Fig. 7B). In addition, we measured total mHtt levels by western blot, and we also observed a significant decrease in mHtt levels, upon AZD8055 treatment in CStS (*Hdh* ^(CAG)150^ 0.2±0.03, *Hdh* ^(CAG)150^ with 0.3 µM AZD8055 0.12±0.02, Fig. 7C). Up until DIV21 (and later) the levels of DARPP-32 and NeuF in *Hdh* ^(CAG)150^ HET CStS are similar to WT, thus, with this model we limited our analysis to mHtt levels. Taken together our results demonstrate that catalytic mTOR inhibition is a potent inducer of neuronal autophagy which leads to a decrease of mHtt levels and to an amelioration of MSNs degeneration.

### Atg4b-dependent Autophagic Flux Modulates HD Progression in vitro

**Figure 8:**
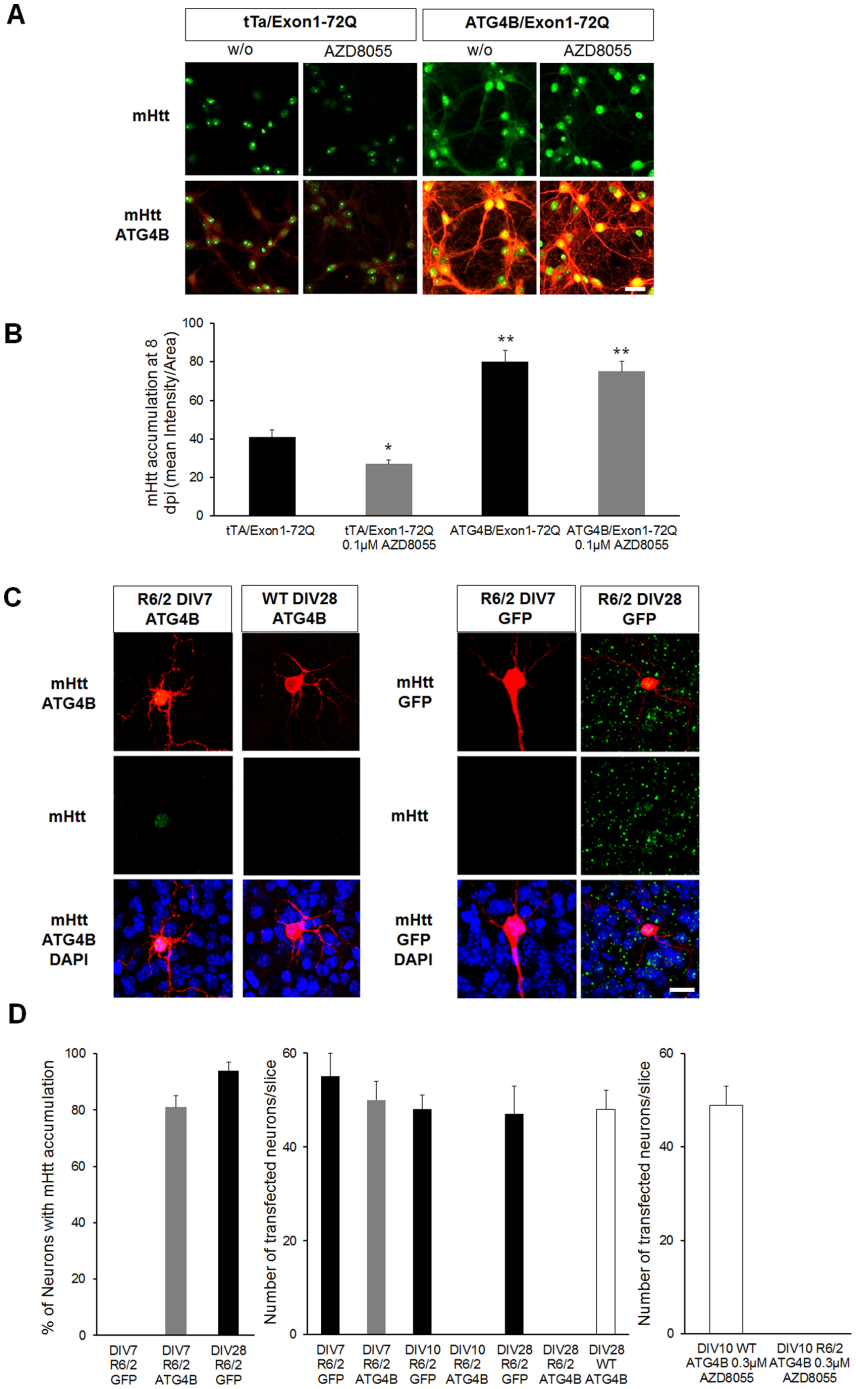
Atg4b dependent-autophagy flux attenuates HD progression. A) Representative images for tTa/Exon1-72Q and Atg4b/Exon1-72Q cortical neurons treated or not with AZD8055 at 8 days post lentiviral infection (dpi). Note the enhanced mHtt accumulation (green) in both Atg4b/Exon1-72Q +/− AZD8055 neurons. B) Quantitative analysis of mHtt intensity per area. Note that AZD8055 reduced mHtt accumulation in tTa/Exon1-72Q neurons but had no effect in Atg4b/Exon1-72Q neurons. C) Representative images of neurons in CStS transfected with gene gun expressing Atg4b or GFP at DIV7 and DIV28. Note how Atg4b accelerates the appearance of mHtt accumulation in neurons of R6/2 slices at DIV7, but it is not inducing toxicity in WT slices. D) Left: quantitative distribution of mHtt accumulation in R6/2 Atg4b and R6/2 GFP expressing neurons at DIV7 and 28. Note how the percentage of mHtt accumulation is almost similar between R6/2 Atg4b neurons at DIV7 and R6/2 GFP neurons at DIV28. Middle: distribution of positive neurons in R6/2 GFP and R6/2 Atg4b slices at DIV 7, 10 and 28. No R6/2 Atg4b neurons were detected at DIV10 and 28. On the other hand WT Atg4b neurons were detected at DIV28, suggesting mHtt-dependent toxicity in the R6/2 Atg4b neurons. Right: AZD8055 is not rescuing the loss of Atg4b neurons in R6/2 slices at DIV10. A) N = 50 images from 3 independent preparations; (C) N = 10 images from 5 independent slices; median values ± SEM; Student’s t test: *p<0.05, **p<0.01 Bars: 10 µm.

To evaluate the impact of Atg4b-dependent autophagic flux in affecting neuronal mHtt accumulation and viability, we co-infected cortical neurons either with tTa/Htt-Exon1-72Q or Atg4b/Htt-Exon1-72Q and analyzed the distribution of mHtt accumulation. In tTa/Htt-Exon1-72Q infected cortical neurons, we observed distinct nuclear inclusions as well as a diffused mHtt distribution. Co-expression of Atg4b and Htt-Exon1-72Q in neurons led to a significant increase of mHtt accumulation, showing that Atg4b-dependent autophagic flux is a central pathway for mHtt degradation and as a result can influence the level of its accumulation (Fig. 8A and Fig. 8B). Remarkably, when we treated tTa/Htt-Exon1-72Q neurons with AZD8055, we observed a decrease of mHtt accumulation. However, a similar AZD8055 application in Atg4b/Htt-Exon1-72Q was unable to revert the mHtt accumulation, suggesting that AZD8055 reduces mHtt levels in an Atg4b-dependent manner (tTa/Htt-Exon1-72Q 41±3.6, tTa/Htt-Exon1-72Q with 0.1 µM AZD8055 27±2.2, Atg4b/Htt-Exon1-72Q 80±2.2, Atg4b/Htt-Exon1-72Q with 0.1 µM AZD8055 75±6.1, Fig. 8B).

To test the effects of Atg4b in CStS, we used gene gun transfections to constitutively express Atg4b or GFP (GFP was used here as a control so that we can detect the few cells that were biollisticaly transfected) in R6/2 CStS (see methods for details). R6/2 CStS exhibit almost no mHtt accumulation by DIV7 which provides an optimal time point to assess changes in mHtt aggregation patterns. By analyzing mHtt distribution in Atg4b and GFP positive neurons, we found a selective appearance of mHtt accumulation in Atg4b positive neurons at DIV7 and loss of Atg4b neurons starting at DIV10 (Fig. 8C, Fig. 8D). At later time points (DIV28) GFP neurons were present and showed similar mHtt accumulation to Atg4b positive cells at DIV7, suggesting neuronal death of Atg4b-positive neurons due to mHtt toxicity (DIV7 R6/2 GFP 55±5, DIV7 R6/2 Atg4b 50±4, DIV10 R6/2 GFP 48±3, DIV10 R6/2 Atg4b 0, DIV28 R6/2 GFP 47±6, DIV28 R6/2 Atg4b 0, Fig. 8C). Remarkably, Atg4b was expressed in WT slices and Atg4b positive cells were still present at DIV28 as we can assess by the number of positive cells that is unchanged, suggesting that these cells are viable during this time (DIV28 WT Atg4b 48±4, Fig. 8C). To assess whether AZD8055 could rescue the loss of Atg4b neurons at DIV10, we treated R6/2 slices for three days from DIV7 to DIV10. We observed no Atg4b positive neurons upon AZD8055 treatment in R6/2 slices, whereas AZD8055 was not altering the number of Atg4b neurons in WT slices, indicating that AZD8055 alleviation of mHtt toxicity is Atg4b-dependent (DIV10 WT Atg4b AZD8055 49±4, DIV10 R6/2 Atg4b AZD8055 0, Fig. 8D). These results in R6/2 slices confirm our earlier observation in cortical neurons and indicate that decreased neuronal Atg4b-dependent autophagic flux accelerates mHtt aggregation in a variety of neuronal cell types. Moreover, we provide evidence that AZD8055 ameliorates HD progression in an Atg4b-dependent manner, demonstrating that inducers of Atg4b-dependent autophagic flux are putative candidates for a disease modifying therapy in HD.

## Discussion

In this study we have investigated the potential of autophagy in preventing HD-associated MSNs degeneration. We established *in vitro* CStS from R6/2 and and Hdh^(CAG)150^ HD mouse models and found that this system recapitulates MSNs degeneration, and thus is suitable for manipulation and monitoring of the HD phenotype specifically in striatal neurons. We then found that enhancement of the autophagy pathway with a potent catalytic mTOR inhibitor, AZD8055, led to increase autophagic flux and blockade of this pathway by overexpressing Atg4b, reversed AZD8055 effects. AZD8055 was shown to ameliorate MSNs degeneration by lowering mHtt accumulation in HD slices. Finally, blockade of Atg4b-dependent autophagic flux was sufficient to accelerate mHtt accumulation and neurodegeneration in slices from a HD mouse model (R6/2), suggesting that this pathway plays a major role in modifying HD onset and progression. Taken together, our results indicate that Atg4b-dependent autophagic flux modifies HD onset and progression. Here we discuss possible implications of these findings and their relationship with previous studies to autophagy, mTOR inhibition and amelioration of HD dependent toxicity in MSNs.

### HD Cortico-striatal Slices Recapitulate MSNs Degeneration

Our analysis of MSNs degeneration using brain slices has revealed the possibility to develop 3D-culture systems, which maintain relevant neuronal subtypes and circuitry as models for neurological disorders. Previous studies using organotypic brain slices from WT, transgenic and mutant mice have shown that neuronal circuits mature and maintain similar *in vivo* characteristics in a dish [7]–[9], [28]. Here we selected two mouse models which develop HD phenotypes in a temporally different manner and assessed if these could be used as an *in vitro* model of HD. The R6/2 mouse expresses a human mutation of exon 1 fragment with 148 to 153 CAG repeats and displays an aggressive HD phenotype that starts at 5 weeks of age, allowing for early assessments of HD phenotype. In contrast to R6/2, the Hdh^(CAG)150^ mouse model has a CAG expansion inserted in the murine HTT gene, under the endogenous promoter and it lacks foreign DNA. This model has a milder HD phenotype that develops later in life allowing us to assess subtler phenotypes [25]. We assessed the development of HD phenotype in slices from R6/2 and Hdh^(CAG)150^. Remarkably we observed that CStS from these mice matured *in vitro* and developed accumulation of mHtt aggregates. In the case of R6/2 CStS this was also accompanied by selective decrease in DARPP-32 and NeuF stainings, indicating that R6/2 CStS are recapitulating major features of the HD-dependent MSNs degeneration and therefore represent a model for HD symptomatic stages. However, in HET *Hdh* ^(CAG)150^ CStS we did not detect an advanced MSNs degeneration (measured as decrease in DARPP-32 and NeuF levels) but rather early signs of mHtt accumulation, showing that HET *Hdh* ^(CAG)150^ CStS mimic HD presymptomatic stages. The combination of these two models allows the manipulation of HD at different progressive states, namely the mechanisms that lead to early accumulation of mHtt as well as later mechanisms that lead to loss of DARPP-32 and NeuF levels. Notably, this is the first study using R6/2 and and Hdh^(CAG)150^ cortico-striatal slices to follow HD-dependent MSNs degeneration for several weeks *in vitro*. Thus, our HD-modeling using CStS represents a novel approach to study the relevance of defined molecular pathways in ameliorating HD onset and progression.

### Conventional Autophagy as a Mechanism to Regulate mHtt Accumulation in Neurons

Autophagy facilitates the clearance of long-lived proteins, aggregates and damaged organelles [34]. This pathway has been widely studied in the context of neurodegenerative disorders (NDs) as a possible mechanism to reduce protein aggregate levels. Indeed, NDs such as Parkinson’s, Alzheimer’s or Huntington’s display common cellular mechanism of protein aggregation and formation of inclusion bodies; and evidence points to a protective role for degradation of protein aggregation [35], [36]. A link between autophagy and HD was prompted by the finding that brains from HD patients exhibited increased autophagic structures [37], [38]. However, evidence for its use in the neurons selectively affected in HD was lacking.

In our study we showed that blockade of Atg4b protease accelerated mHtt accumulation and neurodegeneration, both in primary cultured neurons as well as in CStS from R6/2 mice. These results demonstrate for the first time that conventional neuronal autophagy is a central pathway to alleviate HD progression. In fact, Atg4b not only was found to reduce the LC3 lipidation, but also to enhance p62 accumulation, indicating that Atg4b modulate LC3 autophagosome biosynthesis and cargo recruitment in neurons. Thus, failure to form effective LC3 autophagosome blocked p62 and mHtt degradation, suggesting that Atg4b conventional autophagy regulate mHtt accumulation and toxicity. Moreover, in R6/2 slices, our results demonstrated that Atg4b conventional autophagy is sufficient to enhance mHtt accumulation and death in a variety of neuronal cell types, therefore establishing this pathway as a major modifier for HD progression. Previous results have suggested impairment in cargo recognition for cells expressing mHtt because of the appearance of autophagosomes containing less cytosolic structures than cells expressing Htt [39]. When we analyzed autophagosomes by electron microscopy (EM) in WT and R6/2 slices, we found a comparable number of autophagosomes, containing different cytosolic contents. Moreover, we observed a similar loading of cytosolic structures in WT and R6/2 AZD8055 treated slices, suggesting no cargo recognition failures in R6/2 brain slices. Taken together, our results indicate that Atg4b-dependent autophagic flux is critical to alleviate MSNs degeneration and that autophagosomes contain similar cytosolic contents in WT and R6/2 slices. The differences from our study and previous results most likely lie on the usage of different cell types and HD models, suggesting that mHtt is not blocking cargo recognition in neuronal cells such as MSNs from R6/2 slices. Thus, distinct cargo recognition mechanisms may exist between defined neuronal and non-neuronal cells. This should be an interesting topic for future studies, also in view of the potential therapeutic impact for HD in targeting autophagy in specific neuronal cell populations.

### ATP-competitive mTOR Inhibitors Alleviate MSNs Degeneration by Stimulating Conventional Autophagy

Recent studies have highlighted that mTOR inhibition is inducing autophagy and is lowering mHtt accumulation in non-neuronal cells [16], [20], [30]. We tested the efficiency of an ATP-competitive mTOR inhibitor, AZD8055 in preventing MSNs degeneration in CStS from R6/2 and Hdh^(CAG)150^ mice. We found that AZD8055 treatment rescued the HD phenotype by reducing mHtt aggregate size and density and preventing MSNs degeneration leading to normal DARPP-32 and NeuF levels in R6/2 CStS; and by reducing mHtt accumulation and soluble full-lenght mHtt in HET *Hdh* ^(CAG)150^ mutant CStS. We then dissected the downstream molecular mechanisms of AZD8055-mediated autophagy by blocking LC3 lipidation using Atg4b in cortical neurons. Our results indicated that co-expression of Htt-Exon1-72Q with Atg4b strongly exacerbated the accumulation of mHtt and that under these circumstances AZD8055 was not decreasing mHtt accumulation. Measurements of neuronal autophagic flux using the tandem tagged mCherryGFP-LC3 construct and the degradation of the long-lived protein p62 revealed that AZD8055 was inefficient at inducing flux in the presence of Atg4b. These results demonstrated for the first time that mTOR inhibition induces Atg4b autophagic flux in neurons and that Atg4b-dependent autophagic flux reduces mHtt accumulation in R6/2 and *Hdh* ^(CAG)150^ HET slices and alleviates MSNs degeneration in R6/2 slices. These results bring evidence that conventional autophagy alleviates MSNs degeneration and that ATP-competitive mTOR inhibitors, such as AZD8055, are potent inducers of this pathway in neurons.

### Conclusion

Mutant Htt protein is ubiquitously expressed throughout the body, yet neurons of the cortico-striatal pathway are uniquely sensitive to degeneration, indicating that mechanisms of mHtt-mediated toxicity are different amongst several cell types. Therefore, it is not only important to understand the basic molecular mechanisms of toxicity induced by mHtt but, in particular, study these mechanisms in the neuronal cells mostly affected in this disorder, MSNs. Our results show that HD pathology develops progressively *in vitro* in cortico-striatal slices and that catalytic mTOR inhibitors ameliorate HD phenotype by reducing mHtt accumulation and preventing MSNs degeneration. Moreover we highlighted the importance of Atg4b for neuronal conventional autophagy and open the window for a new potential target in the treatment of HD.

## Materials and Methods

### Mice and Slice Cultures

R6/2 mice expressing human Huntingtin Exon-1Q200 and knock-in *Hdh* ^(CAG)150^ mice expressing endogenous mouse Huntingtin with a (CAG)150 repeat expansion were provided by Gillian Bates (King’s college, London). Both strains were housed in a temperature-controlled room and maintained on a 12 hr light/dark cycle. Food and water were available *ad libitum* and experiments were carried out in accordance with the local authorization guidelines for the care and use of laboratory animals. Slice cultures were established according to the procedure described by Stoppini and colleagues [9], [41] and a horizontal cutting angle of 30° was utilized to produce brain slices with an intact cortico-striatal pathway. Finally, slices were selected, placed on Millicel (Millipore, PICM03050) and cultured in 6-well dishes at 35°C and 5% CO2 in the presence of 1 ml of culture medium. Studies described in this research article were approved by the Swiss cantonal veterinary office and performed according to Novartis animal license number BS-1858.

### Cloning of ATG4B WT and C74A Constructs

Human ATG4B cDNA was obtained from Invitrogen (clone IOH4606). To generate catalytic-inactive ATG4B C74A, QuikChange Mutagenesis Kit (Stratagene) was used with the following primer pair: CCTCGGACACAGGCTGGGGCGCTATGCTGCGGTGTGGACAGAT, ATCTGTCCACACCGCAGCATAGCGCCCCAGCCTGTGTCCGAGG. The cDNAs encoding Atg4B WT or C74A were then HA-tagged by PCR-amplification (primers: ACAGTGGCTAGCCCACCATGGACGCAGCTACTCTGAC, ACTCGAGAATTCTCATGCGTAGTCTGGTACGTCGTACGGATAAAGGGACAGGATTTCAAAGT), inserted into pER3 vector and verified by DNA sequencing.

### Primary Mouse Cortical Neurons Culture and Infections

E16 primary mouse cortical neurons were dissected and cultured as previously described [42]. Primary cortical neurons were then infected 1 day after plating (DIV1) with internally produced lentiviral stocks for tTa (empty backbone), Q72-Htt-Exon1 to model HD progression and mCherry-GFP-LC3, Atg4b WT and Atg4b C74A to study Atg4b conventional autophagic flux, following the published protocol [43]. Viral dose was determined and matched for particle content in ng p24 antigen/ml as measured by ELISA (Zeptometrix Corp, USA).

### Biolistic Transfection

Brain slices were transfected 6 hours after preparation with plasmids encoding ATG4B WT, ATG4B C74A and EGFP using helios gene gun system (Bio-Rad Laboratories, # 165-2431) as previously described [7], [8]. Briefly, 8 µg of plasmid DNA was coated on 4 mg of 1.6 µm diameter gold particles in 20 µl spermidine (250 mM), 27 µl Nupherin (3 mg/ml) and 8 µg DANN. Then the coated DNA was precipitated with 1 mM CaCl_2_, and washed three times in pure ethanol. The gold particle were coated onto PVC tubing, dried using pure N2 gas, and stored at 4°C in dessicant. DNA-coated particles were delivered with a standard pressure of 180 psi (pound per square) and distance of 2 cm from the brain slice tissue. Finally, slices were fixed, stained and analyzed following the protocols described below in the immunohistochemistry and microscopy paragraphs [40].

### Pharmacological Evaluation

Slices were treated with 300 nM AZD8055 (produced by Novartis) from DIV14 to DIV21 by adding a fresh aliquot every second day once the culture medium was exchanged following the same protocol of previous studies [7], [8]. Primary neurons were treated with bafilomycyn A1 (SIGMA, # B1793) and AZD8055 for 24 hours starting at 7 days post infection (dpi).

### Western Blot

Slices and/or primary neurons were washed in PBS and lysed in 1% Triton X-100/PBS containing Complete Mini (Roche, # 04693124001) and PhosSTOP (Roche, # 04906837001). Lysates were ultrasonicated and incubated for B-actin (Sigma, # A5441), pS6 Ser 240/244 (Cell Signalling, # 2215), Akt (Cell Signalling, # 9271), pAKt Ser 473 (Cell Signalling, # 9271), p62 (Lucerna # H00008878-M01), ATG4B (Sigma # A2981) or mutant Htt. (Millipore, # MAB5374, MW1) and Immunoblots were developed with ECL detection reagent (Amersham Biosciences). LC3 (Novus # NB100-2220) was detected in cell lysates using 10% SDS gels and subsequent western blot analysis as previously described [44]. Aggregates were further analysed on agarose gels as described [45]. The mEM48 antibody (Millipore, cat. no.MAB5374) was used in 1% Dry-Milk TBST as primary antibody. An anti-mouse IgG coupled to HRP was diluted 1/10′000 and used as secondary antibody (SIGMA). Immunoblots were developed with ECL detection reagent (Amersham Biosciences).

### Immunohistochemistry

Slices and/or neurons were fixed for 10 minutes in 4% PFA, washed in PBS and blocked for 4 hr at room temperature in 0.3% Triton X-100 20% Horse Serum/PBS (blocking solution). DARPP-32 (Cell Signaling, # 2306S), neurofilament (NeuF, Developmental Studies Hybridoma Bank, University of Iowa; # 2H3), EM48 (Millipore, # MAB5374), VGLUT1 (Millipore # AB5905), Atg4b (Sigma # A2981), NeuN (Millipore, # MAB377) were incubated for 24 hours at 4°C in the blocking solution. Afterwards, slices and/or neurons were washed in PBS, incubated for 2 hrs in 0.3% Triton X-100/PBS with Alexa488 goat anti mouse (life technologies, # A11001), Alexa488 chiken anti mouse (life technologies, # A21200), Alexa555 donkey anti rabbit (life technologies, # A31572), Alexa633 goat anti guine pig (life technologies, # A21105) and Alexa633 goat anti mouse (life technologies, # A21050). Finally, slices and/or neurons were washed in PBS, incubated 10 minutes with DAPI (Invitrogen, # D1306) and embedded on glass dishes using ProLong (Invitrogen, # P36934).

### Microscopy and Quantification

High resolution images were acquired on an upright Zeiss LSM700 confocal microscope, using a Plan-Neofluar 40×/1.3 oil immersion objective. For the quantification of the PolyQ density as well as for DARPP-32, NeuF and mHtt signal intensities, at least ten confocal 3D stacks/slice were acquired in striatum for each experiment (three-five slices per condition), and analyzed using Imaris 7.2 (Bitplane AG) and Image J software. Briefly, single confocal planes of 160 µm×160 µm containing 94±2 dapi positive cells and 70±3 NeuN positive cells were used to measure DARPP-32, NeuF and mHtt intensities as well as PolyQ density and size for the different experiments in slices and fixed brain sections. Total and background intensity levels were measured using Image J and the subtracted values were used for comparison through the different conditions. PolyQ density and size were measured using Imaris 7.2 using the surpass mode and function spot options. Similarly, single confocal planes of 60 µm×60 µm containing 46±5 dapi positive cells and 21±3 NeuN positive cells were used to measure mHtt intensities in primary cortical neurons, as described above.

### Autophagic flux Quantification

Measurements of autophagic flux in mCherry-GFP-LC3 infected cortical neurons were performed using an imaging-based assay as previously reported [23], [31]. In brief, we quantified the total number of autophagic vacuoles (including early autophagosomes and autolysosomes) per cell soma, quantified on the red channel as mCherry was stable both in early autophagosome and autolysosome (Red). As well as number of early autophagosomes alone (Red and Green = yellow), these correspond to early autophagosome because once this organelle fuses with lysosome, the drop in pH quenches GFP fluorescence. The Flux % was then calculated as the ratio of early autophagosomes over total autolysosomes as following:

Flux % = (100– ((Red and Green)/Red) ×100).

We quantified between 30 and 60 cells for each condition. High resolution images were acquired on an upright Zeiss LSM700 confocal microscope using a Plan-Neofluar 40×/1.3 oil immersion objective and dots were analyzed with Imaris 7.2 (Bitplane AG) using the surpass mode and function spot options.

### Electron Microscopy

Corticostriatal slices were fixed by immersion in a fresh fixative (2% paraformaldehyde and 2% EM-grade glutaraldehyde in 0.1 M cacodylate buffer, pH 7.4). Slices still attached to the membrane were rinsed three times in sodium cacodylate buffer (0.1 M, pH 7.4), stained in 1% osmium tetroxide and 1.5% potassium ferrocyanide in 0.1 M cacodylate buffer for 30 minutes followed by 1% osmium tetroxide in water for 30 minutes. After 3 rinses, slices are stained for 45 minutes in 1% uranyl acetate in water. Slices are then dehydrated by steps in ethanol (1×50%, 2×70%, 1×90%, 1×95%, 3×100%). Slices were then embedded in Durcupan resin between 2 foils of Aclar and cured for 24 h at 65°C. After curing, Aclar foils are removed and the region of interest was cut from section, mounted on a block in order to be sectioned at 50 nm thickness. The sections were collected on formvar-coated single-slot grids, stained with uranyl acetate and lead citrate. Images were taken with a side-mounted digital camera (Veleta, Olympus) on a Philips CM10 transmission electron microscopy at 80 kV. The number of autophagosomes was measured in section areas of 10 µm×10 µm and 5 sections/condition were analyzed for mean values.

### Statistical Analysis

All data are expressed as mean ± SEM. Statistical analysis was performed by a Student’s t Test (Excel, Microsoft, USA). The significance level was set at p<0.05.

## References

[1] Vonsattel JP, DiFiglia M (1998) Huntington disease. Journal of neuropathology and experimental neurology 57: 369–384. 9596408 10.1097/00005072-199805000-00001

[2] Nakamura K, Aminoff MJ (2007) Huntington’s disease: clinical characteristics, pathogenesis and therapies. Drugs Today (Barc) 43: 97–9116. 17353947 10.1358/dot.2007.43.2.1050788

[3] Walker FO (2007) Huntington’s disease. Lancet 369: 218–228. 17240289 10.1016/S0140-6736(07)60111-1

[4] Ginovart N, Lundin A, Farde L, Halldin C, Backman L, et al (1997) PET study of the pre- and post-synaptic dopaminergic markers for the neurodegenerative process in Huntington’s disease. Brain 120 (Pt 3): 503–514. 10.1093/brain/120.3.503 9126061

[5] de Almeida LP, Ross CA, Zala D, Aebischer P, Deglon N (2002) Lentiviral-mediated delivery of mutant huntingtin in the striatum of rats induces a selective neuropathology modulated by polyglutamine repeat size, huntingtin expression levels, and protein length. The Journal of neuroscience : the official journal of the Society for Neuroscience 22: 3473–3483. 11978824 10.1523/JNEUROSCI.22-09-03473.2002 PMC6758353

[6] Tabrizi SJ, Reilmann R, Roos RA, Durr A, Leavitt B, et al (2012) Potential endpoints for clinical trials in premanifest and early Huntington’s disease in the TRACK-HD study: analysis of 24 month observational data. Lancet neurology 11: 42–53. 22137354 10.1016/S1474-4422(11)70263-0

[7] Galimberti I, Gogolla N, Alberi S, Santos AF, Muller D, et al (2006) Long-term rearrangements of hippocampal mossy fiber terminal connectivity in the adult regulated by experience. Neuron 50: 749–763. 16731513 10.1016/j.neuron.2006.04.026

[8] Galimberti I, Bednarek E, Donato F, Caroni P (2010) EphA4 signaling in juveniles establishes topographic specificity of structural plasticity in the hippocampus. Neuron 65: 627–642. 20223199 10.1016/j.neuron.2010.02.016

[9] Gogolla N, Galimberti I, DePaola V, Caroni P (2006) Preparation of organotypic hippocampal slice cultures for long-term live imaging. Nat Protoc 1: 1165–1171. 17406399 10.1038/nprot.2006.168

[10] Rubinsztein DC, Marino G, Kroemer G (2011) Autophagy and aging. Cell 146: 682–695. 21884931 10.1016/j.cell.2011.07.030

[11] Wong E, Cuervo AM (2010) Autophagy gone awry in neurodegenerative diseases. Nature neuroscience 13: 805–811. 20581817 10.1038/nn.2575 PMC4038747

[12] Mizushima N, Levine B, Cuervo AM, Klionsky DJ (2008) Autophagy fights disease through cellular self-digestion. Nature 451: 1069–1075. 18305538 10.1038/nature06639 PMC2670399

[13] Codogno P, Mehrpour M, Proikas-Cezanne T (2011) Canonical and non-canonical autophagy: variations on a common theme of self-eating? Nat Rev Mol Cell Biol 13: 7–12. 22166994 10.1038/nrm3249

[14] Fujita N, Hayashi-Nishino M, Fukumoto H, Omori H, Yamamoto A, et al (2008) An Atg4B mutant hampers the lipidation of LC3 paralogues and causes defects in autophagosome closure. Mol Biol Cell 19: 4651–4659. 18768752 10.1091/mbc.E08-03-0312 PMC2575160

[15] Fujita N, Noda T, Yoshimori T (2009) Atg4B(C74A) hampers autophagosome closure: a useful protein for inhibiting autophagy. Autophagy 5: 88–89. 19104152 10.4161/auto.5.1.7183

[16] Jung CH, Ro SH, Cao J, Otto NM, Kim DH (2010) mTOR regulation of autophagy. FEBS Lett 584: 1287–1295. 20083114 10.1016/j.febslet.2010.01.017 PMC2846630

[17] Ravikumar B, Duden R, Rubinsztein DC (2002) Aggregate-prone proteins with polyglutamine and polyalanine expansions are degraded by autophagy. Hum Mol Genet 11: 1107–1117. 11978769 10.1093/hmg/11.9.1107

[18] Ravikumar B, Vacher C, Berger Z, Davies JE, Luo S, et al (2004) Inhibition of mTOR induces autophagy and reduces toxicity of polyglutamine expansions in fly and mouse models of Huntington disease. Nature genetics 36: 585–595. 15146184 10.1038/ng1362

[19] Renna M, Jimenez-Sanchez M, Sarkar S, Rubinsztein DC (2010) Chemical inducers of autophagy that enhance the clearance of mutant proteins in neurodegenerative diseases. J Biol Chem 285: 11061–11067. 20147746 10.1074/jbc.R109.072181 PMC2856980

[20] Roscic A, Baldo B, Crochemore C, Marcellin D, Paganetti P (2011) Induction of autophagy with catalytic mTOR inhibitors reduces huntingtin aggregates in a neuronal cell model. Journal of neurochemistry 119: 398–407. 21854390 10.1111/j.1471-4159.2011.07435.x

[21] Boland B, Kumar A, Lee S, Platt FM, Wegiel J, et al (2008) Autophagy induction and autophagosome clearance in neurons: relationship to autophagic pathology in Alzheimer’s disease. J Neurosci 28: 6926–6937. 18596167 10.1523/JNEUROSCI.0800-08.2008 PMC2676733

[22] Fox JH, Connor T, Chopra V, Dorsey K, Kama JA, et al (2010) The mTOR kinase inhibitor Everolimus decreases S6 kinase phosphorylation but fails to reduce mutant huntingtin levels in brain and is not neuroprotective in the R6/2 mouse model of Huntington’s disease. Molecular neurodegeneration 5: 26. 20569486 10.1186/1750-1326-5-26 PMC2908080

[23] Nyfeler B, Bergman P, Triantafellow E, Wilson CJ, Zhu Y, et al (2011) Relieving autophagy and 4EBP1 from rapamycin resistance. Molecular and cellular biology 31: 2867–2876. 21576371 10.1128/MCB.05430-11 PMC3133392

[24] Davies SW, Turmaine M, Cozens BA, DiFiglia M, Sharp AH, et al (1997) Formation of neuronal intranuclear inclusions underlies the neurological dysfunction in mice transgenic for the HD mutation. Cell 90: 537–548. 9267033 10.1016/s0092-8674(00)80513-9

[25] Heng MY, Detloff PJ, Albin RL (2008) Rodent genetic models of Huntington disease. Neurobiol Dis 32: 1–9. 18638556 10.1016/j.nbd.2008.06.005

[26] Turmaine M, Raza A, Mahal A, Mangiarini L, Bates GP, Davies SW (2000) Nonapoptotic neurodegeneration in a transgenic mouse model of Huntington’s disease. Proc Natl Acad Sci USA 97: 8093–8097. 10869421 10.1073/pnas.110078997 PMC16675

[27] Klapstein GJ, Fisher RS, Zanjani H, Cepeda C, Jokel ES, Chesselet MF, et al (2001) Electrophysiological and morphological changes in striatal spiny neurons in R6/2 Huntington’s disease transgenic mice. J Neurophysiol 86: 2667–2677. 11731527 10.1152/jn.2001.86.6.2667

[28] De Paola V, Arber S, Caroni P (2003) AMPA receptors regulate dynamic equilibrium of presynaptic terminals in mature hippocampal networks. Nat Neurosci 6: 491–500. 12692557 10.1038/nn1046

[29] Horne EA, Coy J, Swinney K, Fung S, Cherry AE, et al (2013) Downregulation of cannabinoid receptor 1 from neuropeptide Y interneurons in the basal ganglia of patients with Huntington’s disease and mouse models. European Journal of Neuroscience 37: 429–440. 23167744 10.1111/ejn.12045 PMC3699342

[30] Ravikumar B, Rubinsztein DC (2006) Role of autophagy in the clearance of mutant huntingtin: a step towards therapy? Mol Aspects Med 27: 520–527. 16973207 10.1016/j.mam.2006.08.008

[31] Nyfeler B, Bergman P, Wilson CJ, Murphy LO (2012) Quantitative visualization of autophagy induction by mTOR inhibitors. Methods Mol Biol 821: 239–250. 22125069 10.1007/978-1-61779-430-8_14

[32] Settembre C, Fraldi A, Jahreiss L, Spampanato C, Venturi C, et al (2008) A block of autophagy in lysosomal storage disorders. Hum Mol Genet 17: 119–129. 17913701 10.1093/hmg/ddm289

[33] Klionsky DJ, Elazar Z, Seglen PO, Rubinsztein DC (2008) Does bafilomycin A1 block the fusion of autophagosomes with lysosomes? Autophagy 4: 849–950. 18758232 10.4161/auto.6845

[34] Sarkar S, Ravikumar B, Rubinsztein DC (2009) Autophagic clearance of aggregate-prone proteins associated with neurodegeneration. Methods Enzymol 453: 83–8110. 19216903 10.1016/S0076-6879(08)04005-6

[35] Ross CA, Poirier MA (2004) Protein aggregation and neurodegenerative disease. Nat Med 10 Suppl: S10–17 10.1038/nm1066 15272267

[36] Soto C (2003) Unfolding the role of protein misfolding in neurodegenerative diseases. Nat Rev Neurosci 4: 49–60. 12511861 10.1038/nrn1007

[37] Banerjee R, Beal MF, Thomas B (2010) Autophagy in neurodegenerative disorders: pathogenic roles and therapeutic implications. Trends Neurosci 33: 541–549. 20947179 10.1016/j.tins.2010.09.001 PMC2981680

[38] Tellez-Nagel I, Johnson AB, Terry RD (1974) Studies on brain biopsies of patients with Huntington’s chorea. J Neuropathol Exp Neurol 33: 308–332. 4150800 10.1097/00005072-197404000-00008

[39] Martinez-Vicente M, Talloczy Z, Wong E, Tang G, Koga H, et al (2010) Cargo recognition failure is responsible for inefficient autophagy in Huntington’s disease. Nat. Neurosci 13(5): 567–76. 10.1038/nn.2528 PMC2860687 20383138

[40] Gogolla N, Galimberti I, DePaola V, Caroni P (2006) Staining protocol for organotypic hippocampal slice cultures. Nature protocols 1: 2452–2456. 17406491 10.1038/nprot.2006.180

[41] Stoppini L, Buchs PA, Muller D (1991) A simple method for organotypic cultures of nervous tissue. J Neurosci Methods 37: 173–182. 1715499 10.1016/0165-0270(91)90128-m

[42] Zafra F, Hengerer B, Leibrock J, Thoenen H, Lindholm D (1990) Activity dependent regulation of BDNF and NGF mRNAs in the rat hippocampus is mediated by non-NMDA glutamate receptors. EMBO J 9: 3545–3550. 2170117 10.1002/j.1460-2075.1990.tb07564.x PMC552104

[43] Regulier E, Zala D, Aebischer P, Deglon N (2004) Lentiviral-mediated gene transfer to model triplet repeat disorders. Methods Mol Biol 277: 199–213. 15201458 10.1385/1-59259-804-8:199

[44] Wiltfang J, Smirnov A, Schnierstein B, Kelemen G, Matthies U, et al (1997) Improved electrophoretic separation and immunoblotting of beta-amyloid (A beta) peptides 1–40, 1–42, and 1–43. Electrophoresis 18: 527–532. 9150936 10.1002/elps.1150180332

[45] Weiss A, Klein C, Woodman B, Sathasivam K, Bibel M, et al (2008) Sensitive biochemical aggregate detection reveals aggregation onset before symptom development in cellular and murine models of Huntington’s disease. J Neurochem 104: 846–858. 17986219 10.1111/j.1471-4159.2007.05032.x

